# The cost of insecurity: from flare-up to control of a major Ebola virus disease hotspot during the outbreak in the Democratic Republic of the Congo, 2019

**DOI:** 10.2807/1560-7917.ES.2020.25.2.1900735

**Published:** 2020-01-16

**Authors:** Thibaut Jombart, Christopher I Jarvis, Samuel Mesfin, Nabil Tabal, Mathias Mossoko, Luigino Minikulu Mpia, Aaron Aruna Abedi, Sonia Chene, Ekokobe Elias Forbin, Marie Roseline D Belizaire, Xavier de Radiguès, Richy Ngombo, Yannick Tutu, Flavio Finger, Madeleine Crowe, W John Edmunds, Justus Nsio, Abdoulaye Yam, Boubacar Diallo, Abdou Salam Gueye, Steve Ahuka-Mundeke, Michel Yao, Ibrahima Socé Fall

**Affiliations:** 1 Department of Infectious Disease Epidemiology, London School of Hygiene and Tropical Medicine, London, United Kingdom; 2 UK Public Health Rapid Support Team, London, United Kingdom; 3 MRC Centre for Global Infectious Disease Analysis, Department of Infectious Disease Epidemiology, School of Public Health, Imperial College London, London, United Kingdom; 4 Global Outbreak Alert and Response Network, Geneva, Switzerland; 5 World Health Organization, Geneva, Switzerland; 6 Ministère de la Santé Publique, Kinshasa, Democratic Republic of the Congo; 7 Institut National de Recherche Biomédicale, Kinshasa, Democratic Republic of the Congo

**Keywords:** outbreak control transmissibility model response viral hemorrhagic fever

## Abstract

The ongoing Ebola outbreak in the eastern Democratic Republic of the Congo is facing unprecedented levels of insecurity and violence. We evaluate the likely impact in terms of added transmissibility and cases of major security incidents in the Butembo coordination hub. We also show that despite this additional burden, an adapted response strategy involving enlarged ring vaccination around clusters of cases and enhanced community engagement managed to bring this main hotspot under control.

Since April 2018, the Democratic Republic of the Congo (DRC) has been facing its largest Ebola virus disease (EVD) outbreak to date, located in the eastern provinces of North Kivu and Ituri. The main hotspot of the epidemic, the Butembo coordination hub, has faced unprecedented violence targeting the response to EVD, which culminated in the destruction of the two EVD treatment centres in February 2019. In this short paper, we evaluate the likely impact of these attacks in terms of added transmissibility and cases, and show how the implementation of a new intervention strategy was followed by the control of the hotspot.

## Epidemiological and security context

The EVD epidemic in West Africa in 2013 to 2016 was a stark reminder of the need for rapid response to and control of emerging pathogen threats [1]. The largest EVD outbreak in recorded history, this terrible epidemic gave public health workers and scientists unprecedented opportunities to deepen their understanding of EVD transmission [2,3] and draw lessons for a better control of future outbreaks [4-6]. Unfortunately since then, the DRC has faced two unrelated EVD outbreaks: in the Equateur province [7], and in the North Kivu and Ituri provinces of Eastern DRC [8].

The ongoing EVD epidemic in North-Kivu and Ituri is the largest in DRC’s history, and so far the second largest worldwide. While transmissibility and mortality of the disease are comparable to previous epidemics [1,8], and despite the availability of experimental vaccine [9] and therapeutics [8], the response has been challenged by the chronic insecurity plaguing the affected area, characterised by the presence of multiple armed groups, extreme poverty, displaced populations and distrust of the government [8]. This context has had two types of effects on the EVD response: added difficulties in carrying out the response activities and direct attacks on the response itself. Since January 2019, more than 350 incidents disrupting the response activities have been recorded, 80% of which were directly targeted at the structures or the personnel of the response. Among these, a third were identified as community resistance to the response activities, which mainly targeted infection prevention and control, safe and dignified burials (SDB) and mixed response teams [10]. Outbursts of extreme violence where responders were directly targeted were also observed, as illustrated during the attacks on the living camp of Biakato Mines and of the coordination office of Mangina on 27 November 2019, which resulted in the death of four responders and one police officer, and in five injured workers [11].

## Three epidemic phases

Previous work has suggested a correlation between insecurity and EVD transmission [12,13], showing that disruptive events were generally followed by increased delays to hospitalisation, lower vaccination effectiveness and increased transmissibility [13]. Here, we quantify the impact of major security incidents, as well as strategic changes to the response, on EVD dynamics in the Butembo coordination hub (Supplementary Figure S1) and estimate the resulting increase in transmissibility and added cases. On 14 August 2019, Butembo was the main hotspot of the epidemic with ca 50% of the confirmed and probable cases (1,426 out of 2,851) and 80% of security incidents (282 of 353) reported. The first key event considered here is the destruction of the Ebola treatment centres (ETC) of Katwa and Butembo (on 24 and 27 February 2019, respectively), which resulted in major disruptions of the response activities. Secondly, a new intervention strategy was implemented on 7 May 2019. Details of this strategy will be described in a forthcoming paper; briefly, its main aims included faster actions (24–48 h) around cases including contact listing, enlarged ring vaccination around clusters of cases, enhanced community-based surveillance, household decontamination and SDB practices. Moreover, decentralisation of response teams to health areas was increased to favour community dialogue and improve acceptance by the communities. We refer to the three resulting time periods as ‘initial’, ‘disrupted’ and ‘control’ phase (Figure panel A).

**Figure:**
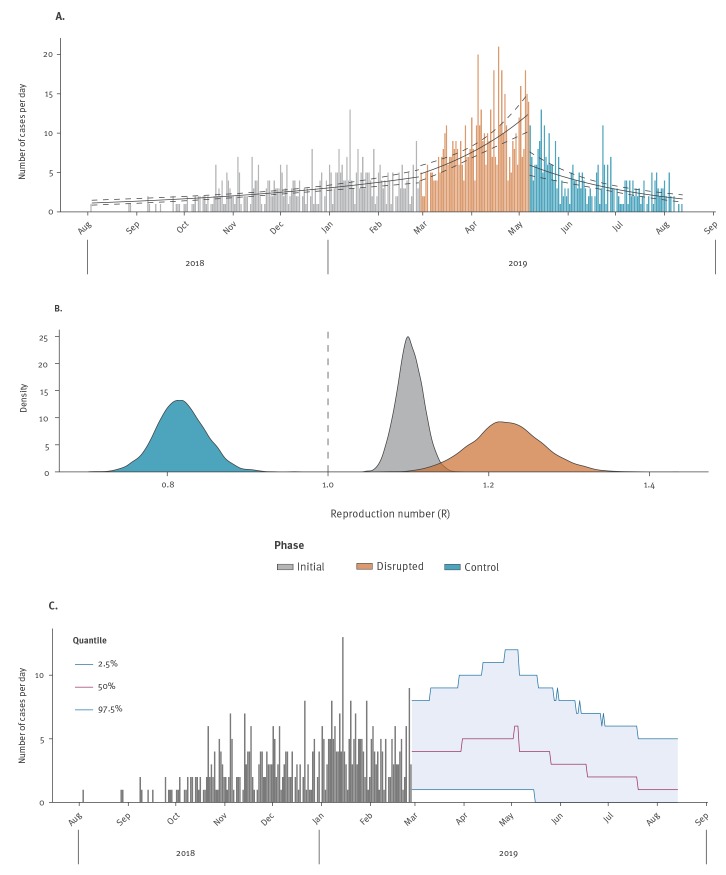
Dynamics of Ebola virus disease in the Butembo coordination hub, Democratic Republic of the Congo, August 2018–September 2019 (n = 1,426)

Initial phase: before the attacks on Ebola treatment centres; Disrupted phase: following attacks; Control phase: following the implementation of the new control strategy.

Panel A: Epidemic curve showing daily numbers of new cases based on dates of symptom onset. Separate log-linear models were calibrated for each time period. Solid lines: estimated average number of daily cases from the models; dashed lines: 95% confidence intervals.

Panel B**:** Distributions of reproduction numbers for the three time periods, estimated from the log-linear models using Wallinga and Lipsitch’s method, using 5,000 samples for each phase.

Panel C: Simulations assuming no increased transmissibility following the attacks, based on 10,000 independent replicates. Red line: median; blue area: 95% credibility interval.

## Estimating transmissibility and added cases

Log-linear models fitted to each phase [14] suggested that while cases were on the rise during the initial phase, a substantial flare-up occurred during the disrupted phase, followed by a trend shift and lasting decrease in incidence during the control phase (Figure panel A). Corresponding distributions of reproduction numbers (average number of secondary cases per case, *R*) were derived using Wallinga and Lipstich’s method [15] after estimating the serial interval distribution from transmission chains (mean: 15.3 days; standard deviation: 7.3 days; see Supplementary Material). Results confirmed significant changes in transmissibility, initially with a median of 1.1 (95% confidence interval (CI): 1.07–1.13). *R* then increased during the disrupted phase to 1.22 (95% CI: 1.14–1.31) before a stark reduction during the control phase, in which all estimated values were less than the control threshold of 1 (median: 0.82; 95% CI: 0.76–0.88). These trend shifts were confirmed by an unsupervised, change point regression method, which identified turning points within 1–2 weeks following the actual dates of the events (Supplementary Material).

The destruction of ETCs in Katwa and Butembo marked the start of a period of heightened insecurity, whose epidemiological impact we can try to quantify. We compared the observed case counts to simulations in which we assumed that the initial phase continued without disruption until the beginning of the control phase. Epidemic trajectories were simulated using the initial distribution of *R* (Figure panel B, green) until 7 May 2019 and then switching to the distribution estimated from the control phase (Figure panel B, blue). Results from 10,000 independent simulations show that while case incidence was expected to increase during the second phase, the flare-up observed during the disrupted phase far exceeded these expectations (Figure panel C). Over the entire time period considered, we estimate that an average of 1,058 (95% CI: 814–1,379) cases would have been generated assuming continued initial phase conditions, to be contrasted with the 1,426 cases actually reported. This suggests the destruction of the ETCs and subsequent disruptions of the response activities such as contact tracing, vaccination, and active case finding, may have caused on average 370 (35%; 95% CI: 46–610) additional cases, corresponding to an expected 250 deaths.

## Discussion

Many aspects of the response, most of which are hard to quantify, probably impacted EVD dynamics in the Butembo coordination hub. Indeed, while representing a major disruption of the response in itself, the destruction of the two ETCs may also be seen as a marker of heightened distrust from the population and overall increased insecurity. Our results suggest this context played a major disruptive role in EVD epidemic control and potentially led to hundreds of additional cases in the affected communities. It is also likely that insecurity during the disrupted phase led to an increase in under-reporting, either through the weakening of passive surveillance (other clinics through which Ebola cases may have transited were also attacked) or the disruption of active case finding. Our results may therefore represent a lower bound for the actual increase in transmissibility and added cases during this time period. 

Importantly, this study also suggests that a change in strategy to adapt to a difficult context can lead to a rapid and drastic reduction in transmissibility, tipping the incidence trends and bringing the outbreak closer to control. At this stage, it is unclear which specific elements drove this reduction in cases, but several indicators suggest a combination of different factors may have played a role. For instance, improved community dialogue permitted better access to health zones, reducing the number of health zones that were inaccessible due to hostile groups from six during the disrupted phase to one during the control phase, and the number of health zones with community resistance from nine to three. Better acceptance probably permitted improvements in all aspects of surveillance and intervention. For example, daily fractions of contacts successfully seen increased from 70% to more than 80%, and a drastic reduction in vaccination gaps was observed: between 22 April and 6 May 2019, 75% (n = 124) of vaccination rings could not be opened within the first 72 h following exposure; in contrast, this number dropped to 12% (n = 68) from 25 May to 8 June 2019. Further modelling work alongside detailed epidemiological and socio-anthropological studies will be needed to disentangle the mechanisms that underpinned these changes and to improve our understanding of the elements key to controlling EVD in highly insecure settings.

## Supplementary Data

688000 Supplement
