# Supplementary material for: The cost of insecurity: from flare-up to control of a major Ebola virus disease hotspot during the outbreak in the Democratic Republic of the Congo, 2019
Source: Euro Surveill. 2020 Jan 16;25(2):1900735. doi: 10.2807/1560-7917.ES.2020.25.2.1900735 (PMC6976886; doi:10.2807/1560-7917.ES.2020.25.2.1900735)
Supplement: Supplement [file 19-00735_JOMBART_Supplement.pdf]

Additional data, methodological information and complementary results

This supplementary material is hosted by Eurosurveillance as supporting information alongside the article "The cost of insecurity: from flare-up to control of a major Ebola virus disease hotspot during the outbreak in the Democratic Republic of the Congo, 2019" on behalf of the authors who remain responsible for the accuracy and appropriateness of the content. The same standards for ethics, copyright, attributions and permissions as for the article apply. Eurosurveillance is not responsible for the maintenance of any links or email addresses provided therein."

For details about the formatting of supplementary material please see our instructions for authors: https://www.eurosurveillance.org/for-authors

BUTEMBO COORDINATION HUB

The Butembo coordination hub is an administrative, geographical unit comprised of 12 health zones: Alimbongo, Biena, Butembo, Kalunguta, Katwa, Kayna, Kyondo, Lubero, Manguredjipa, Masereka, Musienene and Vuhovi. The intensity of transmission in these various zones over the time period considered in our analysis is provided in Figure S1.

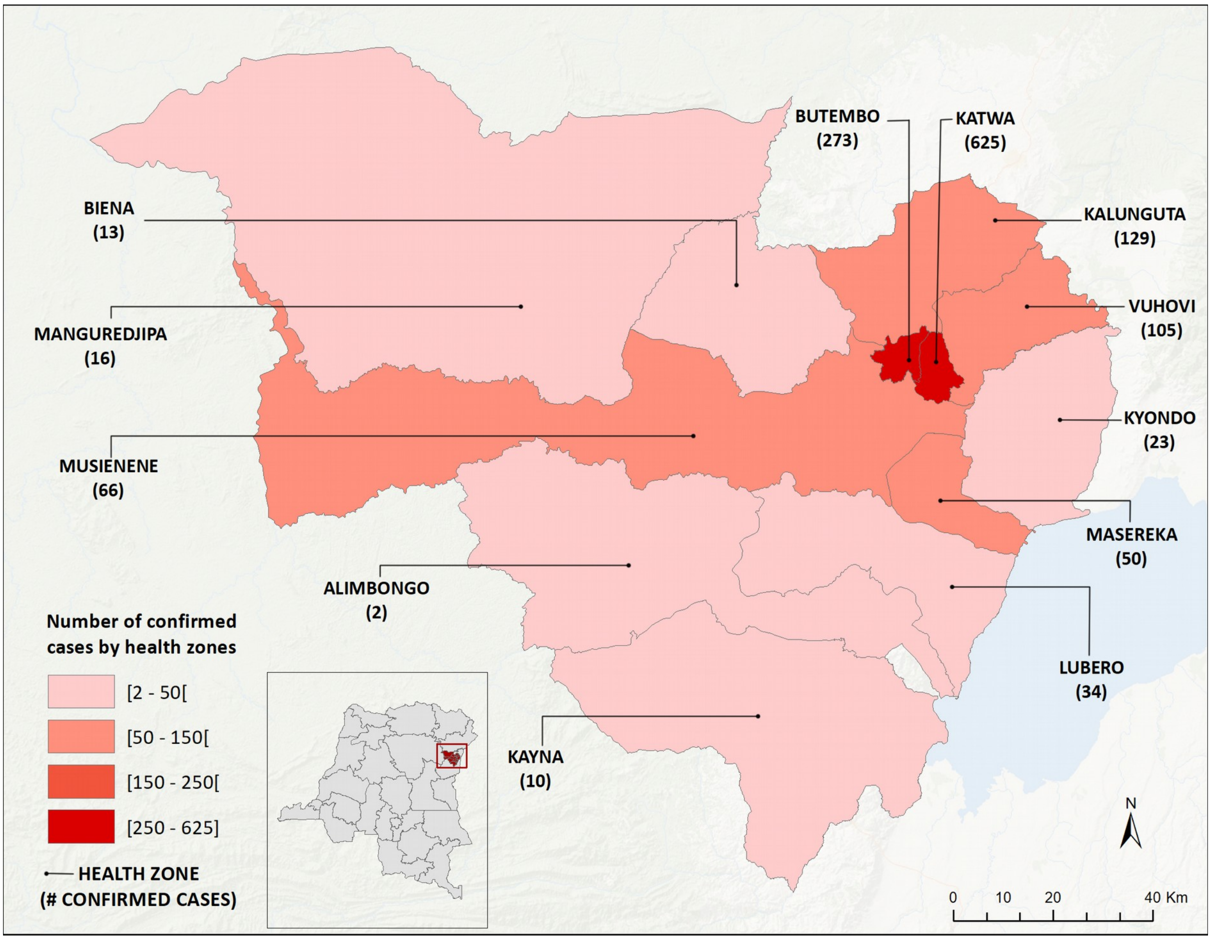

Figure S1: Geographical distribution of confirmed cases notified in the 12 health zones covered by the coordination hug of Butembo, as of 14 August 2019.

ESTIMATION OF TRANSMISSIBILITY

To estimate transmissibility during different time periods, we calibrated separate log-linear models to the number of new daily cases. A log-linear model describes a given time series of cases *y* as a function of time so that:

$$\log(y) = r \times t + \varepsilon$$

where *r* is the daily growth rate of the epidemic, *t* is time, and ε is a normally distributed residual error so that $\varepsilon \sim N(0, s_\varepsilon)$, with s_ε the estimated standard deviation of the residuals. The reproduction number *R* was estimated from the growth rate *r* using Wallinga and Lipsitch's method (1), where *R* is computed as:

$$R = 1 / \int_t e^{-rt}\, g(t)\, dt$$

where g(*t*) is the probability density function of the serial interval distribution. In this study, g(*t*) was estimated using transmission chains of the outbreak, by fitting a Gamma distribution to the delays between dates of onset of primary and secondary cases. To derive samples of values of *R* compatible with the data, samples of *r* were first generated from the log-linear models (using a Student distribution for *r*), and then converted to *R* values using the method described above. All analyses were done using the R software (2), using the RECON packages *incidence* (3) to obtain epidemic curves and fit log-linear models, *epicontacts* (4) to derive the serial interval distribution from transmission chains, and *epitrix* for deriving samples of *R* from *r* (5).

CASE FORECASTING

Case forecasting was achieved using the branching process model implemented in the RECON package *projections* (6) for the R software. Given an epidemic curve y_t which stops at time *t*, the number cases for *t*+1 is drawn from a Poisson distribution such that:

$$y_{t+1} \sim \text{Poisson}(\lambda_t) \quad \text{with} \quad \lambda_t = R \times \sum_{s \leq t} y_s\, w(t - s)$$

where w(.) is the probability mass function of the serial interval distribution (discretized from g()).

Simulated epidemic curves are obtained using the following algorithm:

a) Draw a value of *R* from the estimated distributions (see previous section)
b) Compute the current force of infection λ_t, taken to be the rate of the Poisson distribution
c) Draw cases for the following day y_{t+1} from $y_{t+1} \sim \text{Poisson}(\lambda_t)$
d) Increase *t* by 1 day, go back to a) until all time steps have been simulated

To account for the uncertainty associated with the value of *R* as well as the stochasticity of the Poisson process, 10,000 independent trajectories were simulated.

CHANGE-POINT REGRESSION

A non-parametric, multi-point change-point detection analysis was conducted to assess points during the outbreak where the number of cases per day changed (7). This analysis assumes that there exists a number of periods in the time series considered (here, the epidemic curve) that comes from distinct distributions. For example in a time series with one change point, the values prior to the change point will be distributed according to distribution ‘X1’ and the values after the change point will be distributed according to a seperate distribution ‘X2’. In the non-parametric method used, the form of the distributions is not restricted and the number of change points can be greater than one. Multiple models are fitted to the data assuming different change points and compared using Schwarz information criteria (also known as the Bayesian Information Criteria, BIC) to determine the most likely places where the slope of time series changes (8).

This approach was implemented using the packages *changepoint* and *changepoint.np* (9) for the R software (2). The minimum segment length and the number of quantiles in the empirical distribution were varied to determine the number of change points: the segment length was varied between 1 and 5 weeks, and the number of quantiles was ranged from between 1 and 30 resulting in 150 changepoint analyses. The frequency of the date for the change points were tabulated and the most common change point dates were chosen.

The change point analysis returned 3 dates with greater frequency than any other dates. These dates were 2018-12-24, 2019-03-04, and 2019-05-20 (Figure S2). Each of these dates occurred in roughly 16-20% of change point models, with the next most frequent being in 10% of models. The first date coincides with the election period during which operations were halted for a week. The last two dates are consistent with the changes in time periods in the main analysis, providing an independent indication of changes in the number of cases in the outbreak.

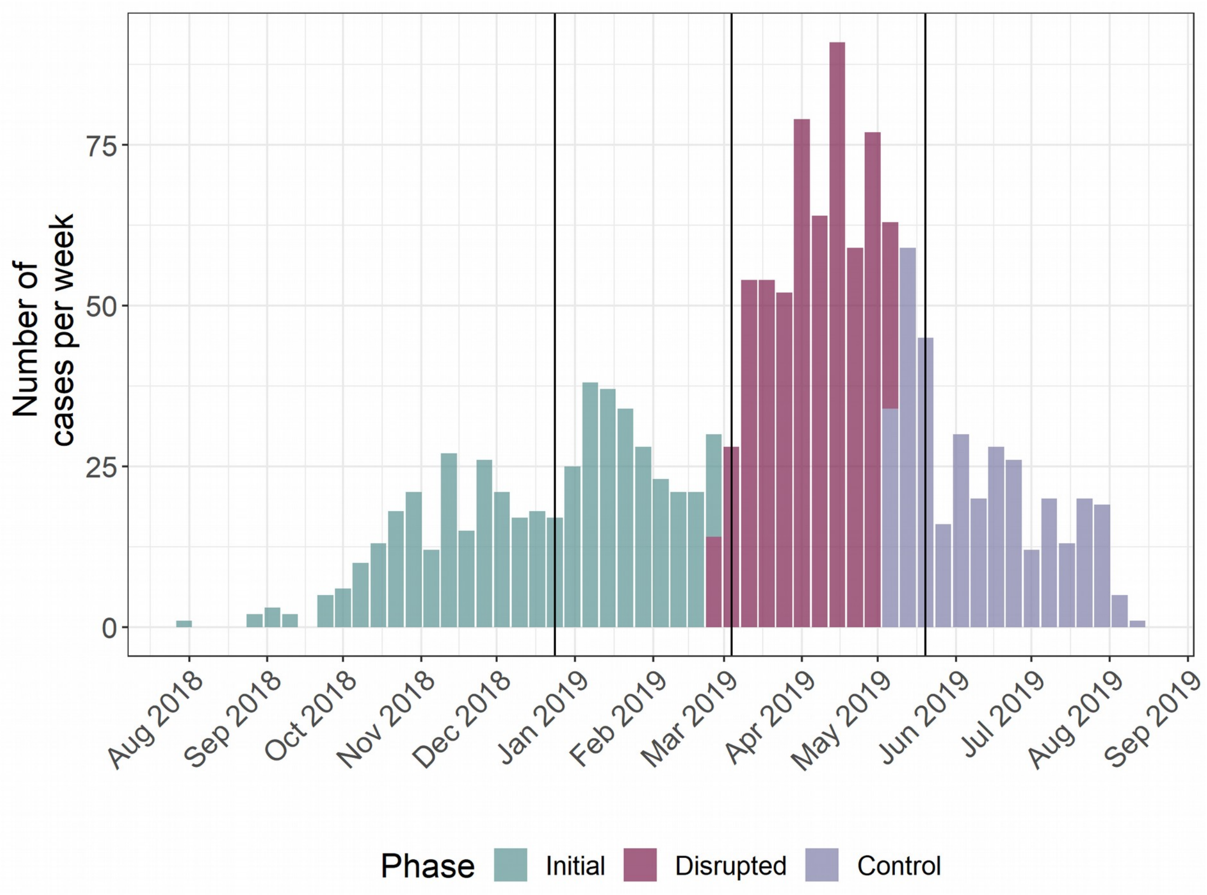

Figure S2: Change point analysis compared to the number of cases of Ebola per week over time. The three vertical lines represent the days which were estimated as change points from the data.

REFERENCES

1. Wallinga J, Lipsitch M. How generation intervals shape the relationship between growth rates and reproductive numbers. Proc Biol Sci. 2007 Feb 22;274(1609):599–604.

2. R Core Team. R: A Language and Environment for Statistical Computing [Internet]. Vienna, Austria: R Foundation for Statistical Computing; 2019. Available from: https://www.R-project.org/

3. Kamvar ZN, Cai J, Pulliam JRC, Schumacher J, Jombart T. Epidemic curves made easy using the R package *incidence*. F1000Res. 2019 Jan 31;8:139.

4. Nagraj VP, Randhawa N, Campbell F, Crellen T, Sudre B, Jombart T. epicontacts: Handling, visualisation and analysis of epidemiological contacts. F1000Res [Internet]. 2018 May 10 [cited 2018 Jul 13];7. Available from: https://f1000research.com/articles/7-566/v1/pdf

5. Small Helpers and Tricks for Epidemics Analysis • epitrix [Internet]. [cited 2018 Feb 5]. Available from: http://www.repidemicsconsortium.org/epitrix/

6. Project Future Case Incidence [Internet]. [cited 2019 Sep 16]. Available from: http://www.repidemicsconsortium.org/projections/

7. Zou C, Yin G, Feng L, Wang Z. Nonparametric maximum likelihood approach to multiple change-point problems. Ann Stat. 2014 Jun;42(3):970–1002.

8. Haynes K, Fearnhead P, Eckley IA. A computationally efficient nonparametric approach for changepoint detection. Stat Comput. 2017 Sep 1;27(5):1293–305.

9. Killick R, Eckley I. changepoint: An R package for changepoint analysis. J Stat Softw. 2014;58(3):1–19.
